# Real-World Systemic Treatment Patterns, Survival Outcomes, and Prognostic Factors in Advanced Hepatocellular Carcinoma: A 15-Year Experience from a Low-Resource Setting

**DOI:** 10.3390/cancers17172729

**Published:** 2025-08-22

**Authors:** Jirapat Wonglhow, Chirawadee Sathitruangsak, Patrapim Sunpaweravong, Panu Wetwittayakhlang, Arunee Dechaphunkul

**Affiliations:** 1 Division of Medical Oncology, Department of Internal Medicine, Faculty of Medicine, Prince of Songkla University, Songkhla 90110, Thailand; sjirawadee@gmail.com (C.S.); spatrapi@medicine.psu.ac.th (P.S.); dr.arunee@gmail.com (A.D.); 2 Gastroenterology and Hepatology Unit, Division of Internal Medicine, Faculty of Medicine, Prince of Songkla University, Songkhla 90110, Thailand; wet.panu@gmail.com

**Keywords:** hepatocellular carcinoma, treatment sequence, targeted therapy, immunotherapy, chemotherapy, prognostic factor, survival, systemic treatment

## Abstract

**Background:** The treatment landscape for advanced hepatocellular carcinoma (HCC) has evolved significantly recently; however, access to novel agents remains limited because of high costs. This study aimed to evaluate the systemic treatment patterns and survival outcomes for advanced HCC across different systemic treatment sequences under real-world resource constraints. **Methods:** This retrospective study was conducted at a tertiary center in Southern Thailand. The medical records of patients (*n* = 330) with advanced HCC treated with systemic therapy between 2010 and 2024 were reviewed. Outcomes included overall survival (OS), progression-free survival (PFS), and objective response rate (ORR). Prognostic factors for OS were investigated. **Results:** First-line therapies included tyrosine kinase inhibitor (TKI; 69.7%), chemotherapy (23.3%), immunotherapy (IO)/targeted therapy (3.6%), dual IO (1.8%), and IO monotherapy (1.5%). The median OS, PFS, and ORR for each cohort were 7.2, 5.2, 10.9, 8.5, and 8.6 months; 3.94, 3.22, 3.48, 6.19, and 2.69 months; and 9.6%, 10.4%, 16.7%, 0%, and 20.0%, respectively. OS improved with increasing lines of therapy (4.5, 12.2, 19.4, and 40.7 months for one to four lines, respectively). Portal vein tumor thrombus, ascites, elevated bilirubin level, high alpha-fetoprotein level, and poor Eastern Cooperative Oncology Group performance status were associated with poor prognosis; multiple treatment lines and overweight status were associated with improved OS. **Conclusions:** In this large real-world cohort, TKIs remained the mainstay effective treatment option because of limited access to IO-based regimens. Sequential systemic therapy significantly improved survival, emphasizing the importance of preserving treatment eligibility and multidisciplinary team involvement. Chemotherapy could be considered a viable option in resource-limited settings.

## Simple Summary

Hepatocellular carcinoma, the most common type of liver cancer, is a serious condition that often requires specific systemic treatment in its advanced stages. The selection of appropriate treatment is complicated and depends on various factors, such as liver function, overall patient condition, and cost. In many countries, including Thailand, access to newer treatments such as immunotherapy is limited by high costs. In this study, we reviewed 15 years of real-world data from a hospital in Southern Thailand to assess treatment patterns and patient survival. We found that receiving more than one line of treatment, regardless of the pattern, improved patient survival. Targeted therapy remained beneficial when immunotherapy was unavailable. Chemotherapy also provided clinical value. These findings highlight the importance of maintaining patients’ clinical status to enable subsequent treatment and close collaboration with healthcare teams, especially in settings where access to newer drugs is limited.

## 1. Introduction

Hepatocellular carcinoma (HCC) is a leading cause of cancer-related mortality, accounting for over 800,000 deaths globally each year and ranking as the third most common cause of cancer-related deaths worldwide [1]. HCC arises from various etiologies, most notably viral infections, mainly hepatitis B virus (HBV) and hepatitis C virus (HCV) infections, as well as non-viral factors such as alcoholic and non-alcoholic steatohepatitis [2]. Treatment selection for advanced-stage HCC is complex and depends on multiple factors, including tumor characteristics and staging, liver function, and patient fitness [3].

Historically, the tyrosine kinase inhibitor (TKI) sorafenib was the first and mainstay drug for patients with advanced HCC who were ineligible for locoregional therapies [4,5]. Lenvatinib, another TKI, was approved for first-line treatment approximately 10 years later and demonstrated non-inferior overall survival (OS) compared with sorafenib [6]. When choosing between these two drugs, clinicians should consider their slightly different toxicity profiles and the better progression-free survival (PFS) and objective response rate (ORR) associated with lenvatinib, possibly reflecting its higher potency [4,5,6,7]. More recently, immunotherapy (IO) has also transformed the therapeutic landscape, with the approval of atezolizumab plus bevacizumab and durvalumab plus tremelimumab providing new first-line options that improve survival and expand treatment options for patients with HCC [8,9,10].

Despite these advancements, significant uncertainty remains regarding the optimal sequencing of these therapies. To date, sorafenib is the only regimen with approved second-line treatment options following its use [11], including cabozantinib, regorafenib, ramucirumab, nivolumab, and pembrolizumab [12,13,14,15,16,17,18]. However, evidence regarding effective sequencing strategies post-IO, and even after lenvatinib treatment, is limited. No standard second-line treatment currently exists, and evidence supporting subsequent therapy options is lacking, presenting a critical gap in the management of advanced HCC. Moreover, the cost of atezolizumab plus bevacizumab and durvalumab plus tremelimumab regimens remains high and limits access in many countries. In Thailand, TKIs continue to be the main treatment option. Chemotherapy (CMT) regimens, such as 5-fluorouracil, leucovorin, and oxaliplatin (FOLFOX), although not approved worldwide, are used in countries where TKIs and IO are inaccessible and have shown some benefit [19,20,21].

Considering the rapid evolution of systemic therapies for advanced HCC, evaluating and selecting the optimal treatment sequence have become increasingly challenging but essential for maximizing patient survival, especially in resource-limited countries. Treatment decisions are no longer solely guided by disease stage; rather, they require multidisciplinary input and a tailored approach that considers individual patient characteristics. This study aimed to evaluate systemic treatment patterns and survival outcomes across different systemic treatment sequences for patients with advanced HCC under real-world resource constraints.

## 2. Materials and Methods

### 2.1. Study Participants

This retrospective cohort study included patients diagnosed with advanced HCC between January 2010 and December 2024 at Songklanagarind Hospital, Songkhla, Thailand. Patients were considered eligible if they met the following inclusion criteria: (1) a confirmed diagnosis of HCC based on either histopathological findings or typical imaging features (arterial phase hyperenhancement with washout in the portal venous or delayed phase); (2) advanced-stage disease, characterized by progression following/resistance to local therapies, presence of portal vein tumor thrombus (PVTT), or evidence of metastasis; (3) age ≥ 18 years; and (4) receipt of palliative first-line systemic treatment, including IO, targeted therapy, or CMT. Patients with a concurrent diagnosis of another malignancy were excluded.

Some patients with Barcelona Clinic Liver Cancer (BCLC) stage-B disease were treated with systemic therapy instead of locoregional modalities if they were unsuitable for surgical resection, transplantation, or transarterial chemoembolization (TACE), due to factors such as large or bilobar tumors, diffuse infiltration, PVTT, poor hepatic reserve, or prior locoregional therapy failure.

Baseline data regarding age, sex, Eastern Cooperative Oncology Group (ECOG) performance status (PS), body mass index (BMI), presence and etiology of cirrhosis, and initial laboratory test results were extracted from the hospital’s electronic medical record system. Information on treatment modalities, including initial systemic therapy, concurrent treatment, and subsequent lines of therapy, and tumor-related characteristics, such as BCLC stage, number and size of intrahepatic lesions, alpha-fetoprotein (AFP) level, and presence of PVTT, extrahepatic metastasis, and ascites, was also obtained.

### 2.2. Study Procedures

All included patients underwent one of several first-line systemic therapies, including atezolizumab plus bevacizumab, durvalumab plus tremelimumab, nivolumab, lenvatinib, sorafenib, FOLFOX, and doxorubicin therapy. In patients without access to reimbursable TKIs and IO-based therapies due to criteria restrictions or financial constraints, CMT was offered as an alternative first-line regimen. Each regimen was administered according to the standard dosing and schedule. Atezolizumab (1200 mg) was delivered intravenously (IV) over 60 min followed by bevacizumab (15 mg/kg IV over 90 min) once every 3 weeks. Durvalumab (1500 mg IV) was administered once every 4 weeks, with a single induction dose of tremelimumab (300 mg IV) administered on the first day of the first cycle. Nivolumab was administered at 240 mg IV biweekly. Lenvatinib was administered orally once daily (12 mg for patients weighing ≥60 kg; 8 mg for those weighing <60 kg). Sorafenib (400 mg) was administered orally twice daily. For the FOLFOX regimen, oxaliplatin (85 mg/m^2^ IV), leucovorin (400 mg/m^2^ IV), and 5-fluorouracil (400 mg/m^2^ IV bolus and then 2400 mg/m^2^ via continuous infusion over 46 h) were administered once every 2 weeks. Doxorubicin (60–75 mg/m^2^ IV) was administered every 3 weeks.

Treatment was continued until radiographic or clinical disease progression, unacceptable toxicity, patient death, or discontinuation by patient choice. The dose was adjusted at the discretion of the treating oncologist based on ECOG PS and laboratory test results. In cases in which first-line therapy failed or was no longer tolerated, treatment decisions regarding second-line and later regimens were considered and individualized according to drug accessibility, patient clinical condition, and personal preferences.

### 2.3. Endpoints

The primary endpoint was OS, assessed across various systemic treatment patterns. The secondary endpoints were PFS and ORR to first-line therapy. Clinical prognostic factors associated with OS were also identified.

OS was defined as the interval between the initiation of first-line systemic treatment and death from any cause. PFS was measured from the date of initiation of the first treatment cycle until either radiological disease progression or death, whichever occurred first. ORR was evaluated using the Response Evaluation Criteria in Solid Tumors (RECIST) version 1.1 based on serial radiological assessments. To monitor treatment response, patients underwent chest and abdominal computed tomography approximately every 2 to 3 months throughout the course of therapy.

### 2.4. Statistical Analysis

Continuous variables are reported as median and interquartile range (IQR) or mean and standard deviation (SD), depending on the data distribution. Categorical variables are presented as count and percentage. Patterns of systemic treatment were visualized using a Sankey diagram to illustrate flow across different lines of therapy. OS and PFS were estimated using the Kaplan–Meier method, and differences between groups were assessed using log-rank test. Univariate Cox proportional hazards regression was used to assess the factors associated with OS, and potentially significant variables were then included in multivariate Cox regression analysis to identify independent prognostic factors.

R version 4.3.1 (R Foundation for Statistical Computing, Vienna, Austria) was used for all statistical analyses. Statistical significance was set at a two-sided *p* value of less than 0.05.

## 3. Results

### 3.1. Patient Characteristics

In total, 330 patients diagnosed with advanced HCC were included in the analysis, of which 230 (69.7%) received a TKI as first-line systemic therapy. CMT was administered to 77 patients (23.3%), whereas 12 patients (3.6%) underwent a combination of IO and targeted therapy, 6 (1.8%) underwent dual IO, and 5 (1.5%) underwent single-agent IO. The baseline clinical characteristics across treatment cohorts are summarized in Table 1. Patients in the CMT group tended to be younger than those in the other cohorts. The dual IO and IO-monotherapy groups had higher proportions of patients with ECOG PS 2 and ascites. Furthermore, patients receiving TKIs or IO/targeted therapy as first-line treatment were more likely to have a Child–Turcotte–Pugh (CTP) class A designation than patients in the CMT, dual IO, or IO-monotherapy cohorts.

### 3.2. Treatment Pattern and Information

**Figure 1:**
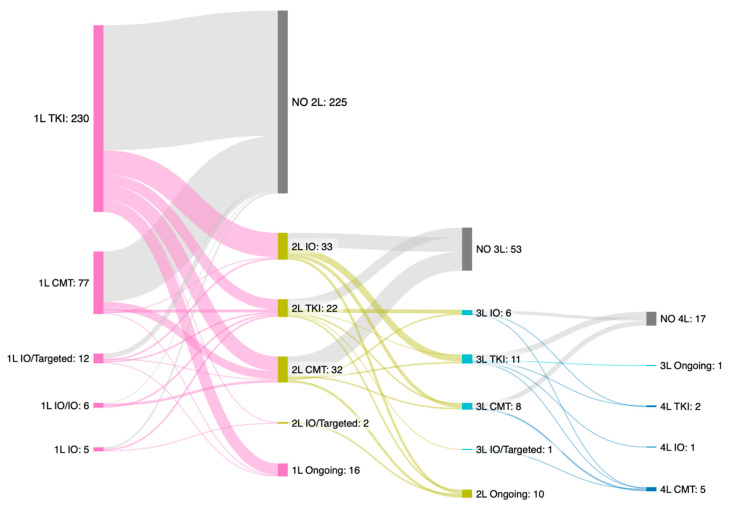
Treatment pattern sequencing of systemic treatments in advanced hepatocellular carcinoma. TKI, tyrosine kinase inhibitor; CMT, chemotherapy; IO, immunotherapy; Targeted, targeted therapy; 1L, first-line treatment; 2L, second-line treatment; 3L, third-line treatment; 4L, fourth-line treatment.

Most patients who received TKIs (73.0%), CMT (81.8%), or IO/targeted therapy (58.4%) as first-line treatment underwent only a single line of systemic therapy. Overall, 26.9% of all patients underwent second-line treatment, whereas only 7.9% underwent third-line therapy. The proportions of patients in the TKI, CMT, IO/targeted therapy, dual IO, and IO-monotherapy cohorts who advanced beyond first-line treatment were 26.9%, 18.2%, 41.7%, 83.3%, and 60.0%, respectively (Supplementary Table S1). The detailed sequences of first-line and subsequent treatments are illustrated in Figure 1 and Supplementary Table S2. The most frequently observed treatment pattern was a single line of TKI treatment, followed by a single line of CMT. Other common patterns included first-line TKI treatment, followed by IO or CMT as the second-line treatment.

### 3.3. OS

**Figure 2:**
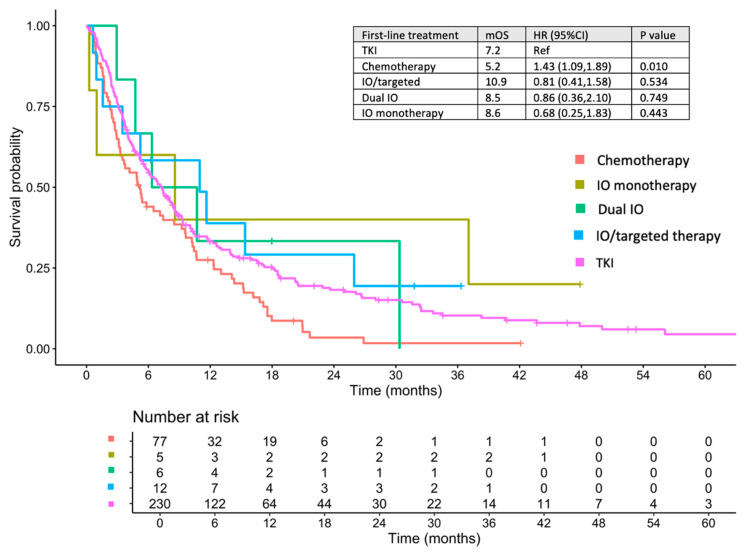
Overall survival of patients treated with systemic therapy stratified by first-line systemic therapy. TKI, tyrosine kinase inhibitor; IO, immunotherapy; HR, hazard ratio; CI, confidence interval; mOS, median overall survival; Ref, reference value.

The median follow-up duration for the entire cohort was 6.2 months (range, 0.03–81.34 months). At the data cut-off date (30 April 2025), 284 patients (86.1%) had died. Median follow-up durations for the first-line TKI, CMT, IO/targeted therapy, dual IO, and IO-monotherapy cohorts were 6.8, 5.0, 8.6, 8.5, and 8.6 months, respectively. Median OS for the entire population was 6.8 months (95% confidence interval [CI], 5.5–8.3). Stratified by first-line treatment, the median OS rates were 7.2, 5.2, 10.9, 8.5, and 8.6 months for TKI therapy, CMT, IO/targeted therapy, dual IO, and IO monotherapy, respectively (Figure 2). Additional OS data for individual regimens is presented in Supplementary Figure S1. In the entire cohort, only three patients (0.9%) survived beyond 5 years; their detailed baseline characteristics and treatment course are provided in Supplementary Table S3.

**Figure 3:**
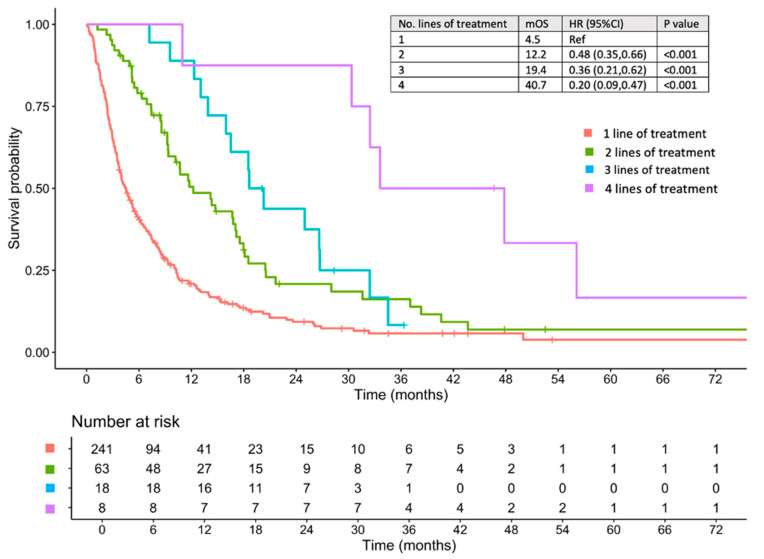
Overall survival of patients treated with systemic therapy stratified by number of systemic treatment lines received. HR, hazard ratio; CI, confidence interval; mOS, median overall survival; Ref, reference value.

When analyzed by the number of systemic treatment lines, the median OS rates were 4.5, 12.2, 19.4, and 40.7 months for patients who underwent one, two, three, and four lines of therapy, respectively (Figure 3). The median OS for each treatment pattern in first-line and second-line treatments is presented in Table 2.

Among patients who underwent first-line TKI therapy, those who underwent second-line treatment had improved outcomes—median OS was 33.07, 18.53, and 13.08 months with TKI, IO, and CMT, respectively (Supplementary Figure S2). One patient underwent IO/targeted therapy as second-line treatment and remained on therapy for 7.72 months of follow-up. For patients starting with CMT, subsequent treatment with TKI, IO, or CMT yielded a median OS of 12.32, 5.82, and 15.56 months, respectively (Supplementary Figure S3).

In the subgroup of 13 patients who received TKIs as both first- and second-line therapies, the median OS was 33.1 months (95% CI, 16.7–NA). Most patients were men (84.6%), <65 years old (69.2%), with Child–Pugh class A liver function (92.7%) and tumor largest diameter <5 cm. (69.2%). All patients had ECOG PS 0–1 at first-line initiation. The most common etiology of liver disease was HBV (46.2%) and HCV (46.2%) infections. The majority had BCLC stage C disease (69.2%) without extrahepatic metastases at baseline (61.5%). The median interval between first-line initiation and progression was 11.5 months.

Regarding CTP class, median OS of CTP class A patients was significantly longer than that of CTP class B patients (8.51 vs. 3.25 months; hazard ratio [HR] 0.39, 95% CI 0.29–0.52; *p* < 0.001). In the CTP class A subgroup, patients treated with TKI, CMT, IO/targeted therapy, dual IO, and IO monotherapy had a median OS of 8.25, 9.23, 11.63, 20.53, and 37.06 months, respectively (Supplementary Figure S4); the corresponding OS for CTP class B patients was 3.68, 2.74, 1.58, 5.54, and 0.99 months, respectively (Supplementary Figure S5).

### 3.4. PFS

The overall median PFS for the study population was 3.68 months (95% CI, 3.09–4.34). When stratified by first-line systemic therapy, the median PFS rates for TKI, CMT, IO/targeted therapy, dual IO, and IO monotherapy were 3.94, 3.22, 3.48, 6.19, and 2.69 months, respectively (Supplementary Figure S6). Further details regarding each regimen are provided in Supplementary Table S4.

Subgroup analysis by CTP class showed that CTP class A patients had significantly longer PFS than CTP class B patients (4.20 vs. 2.46 months; HR 0.57, 95% CI 0.43–0.75; *p* < 0.001). Median PFS for CTP class A patients by first-line treatment was 4.11, 3.75, 4.37, 12.91, and 23.39 months for TKI, CMT, IO/targeted therapy, dual IO, and IO monotherapy, respectively (Supplementary Figure S7); the corresponding PFS for CTP class B patients was 2.63, 2.45, 1.58, 2.27, and 0.26 months, respectively (Supplementary Figure S8).

### 3.5. ORR

Approximately one-third of all patients (108/330) did not undergo response evaluation. The ORRs in the first-line treatment cohorts for TKI, CMT, IO/targeted therapy, dual IO, and IO monotherapy were 9.6%, 10.4%, 16.7%, 0%, and 20.0%, respectively (Supplementary Table S5). Among patients who achieved complete response (CR), one was in the IO-monotherapy group. This patient, categorized as BCLC stage C owing to PVTT without distant metastasis, had a favorable response after treatment. Although atezolizumab/bevacizumab was initially planned, recent upper gastrointestinal bleeding from esophageal varices necessitated switching to IO monotherapy (nivolumab). Following systemic therapy with partial response, TACE was performed, ultimately resulting in CR. Another patient who achieved CR was in the TKI cohort. This patient also had BCLC stage-C disease with PVTT, but no extrahepatic spread. The patient received sorafenib, followed by external beam radiotherapy (EBRT) targeting the PVTT and subsequent TACE, which led to CR. Details of the individual regimens used in the TKI and CMT subgroups are provided in Supplementary Table S6.

### 3.6. Prognostic Factors for OS

Multivariate analysis identified PVTT, ascites, elevated total bilirubin (≥2 mg/dL), high AFP level (≥200 ng/dL), and poor ECOG PS as factors significantly associated with shorter OS. Notably, the type of first-line systemic therapy was not significantly correlated with survival outcomes. Conversely, the multiple lines of systemic treatment and overweight status were key favorable prognostic factors and were both independently associated with improved OS (Table 3).

## 4. Discussion

This retrospective study provides a comprehensive overview of real-world systemic treatment patterns and survival outcomes in advanced HCC over the past 15 years at a tertiary referral center in Southern Thailand. Our findings offer important insights into treatment access, sequencing, and outcomes across various regimens, particularly in resource-limited settings where TKIs remain the mainstay treatment and CMT serves as an alternative for patients who are unable to access standard therapies. Only a small proportion of patients received IO-based therapies as first-line treatment, primarily because of their recent introduction and high costs. Notably, only one-fourth of the cohort underwent second-line treatment. The most common treatment sequence was first-line TKI treatment followed by second-line IO monotherapy, followed by TKI-CMT sequences. However, the ability to receive second-line treatment had a greater impact on OS than the specific treatment sequence.

Sorafenib was the most frequently used first-line agent, reflecting historical practice patterns and reimbursement limitations in Thailand [22]. In the Thai healthcare system, only patients covered under the Civil Servant Medical Benefit Scheme (CSMBS) are eligible for government-funded first-line standard therapy. Sorafenib has been the only reimbursable agent since 2019 under specific criteria, whereas lenvatinib became reimbursable only in 2023. Throughout the study period, CMT was often offered to patients without access to targeted therapy, and IO-based therapies have been used only in the past 1–2 years, primarily for patients with out-of-pocket payment. Therefore, the predominance of sorafenib use is largely attributable to reimbursement constraints rather than shifts in clinical practice or efficacy perceptions, and the influence of historical treatment periods on OS is possibly minimal. Although IO-based regimens, such as atezolizumab plus bevacizumab and durvalumab plus tremelimumab, are now considered the standard of care globally [3,8,9,10], their use in this cohort was limited owing to accessibility barriers. Although not standard in Western practice, CMT continues to be utilized in resource-constrained Asian settings, which is consistent with the findings of previous studies [19,20,21]. Data on second-line treatments are heterogeneous and mainly based on evidence following sorafenib treatment, making direct comparisons across regimens difficult. Therefore, this study captured the diversity and complexity of real-world treatment patterns and emphasized the need for adaptable treatment strategies tailored to both clinical and socioeconomic contexts.

The median OS in each treatment cohort was lower than those reported in several pivotal clinical trials [4,5,6,8,9,10] but comparable to outcomes in some prior real-world studies [23,24,25]. These differences may be attributed to the inclusion of patients with CTP class B and poor ECOG PS, who were excluded from the clinical trials. Only 18% of patients received second-line therapy, likely contributing to the relatively short median OS and highlighting the need to improve access to subsequent treatments. TKIs, particularly sorafenib, yielded a median OS of 7.2 months, consistent with the outcomes of earlier Asian trials [5]. The median OS for CMT was 5.2 months, which increased to 9.2 months for CTP class A patients. These findings further support the use of CMT as a reasonable alternative for selected patients with preserved liver function but no access to TKIs or IO-based treatments [19]. However, the median OS from first-line CMT was still significantly inferior to that of the TKI group. This difference possibly reflects a combination of treatment effect and real-world selection factors: CMT was more commonly used in patients ineligible for reimbursable TKIs/IO or with poorer baseline status, which may confound comparisons in this non-randomized setting. As shown in Table 2, even among patients starting with CMT, those who were able to proceed to second-line therapy achieved markedly longer OS. Consistent with this, our analyses emphasize that maintaining PS and liver function to enable sequential lines of therapy is a key driver of survival. However, the findings related to IO-based regimens should be interpreted with caution because of small sample sizes.

Access to second-line therapy in Thailand is severely limited, even as multiple treatment options and sequencing strategies continue to be evaluated globally [26]. Nivolumab, approved for use after sorafenib failure, is the only second-line agent reimbursed under the CSMBS scheme, with eligibility restricted to those with CTP class A and ECOG PS 0–1. Consequently, most patients in the first-line TKI cohort who did not proceed to second-line therapy were ineligible due to poor PS and/or impaired liver function. In non-TKI cohorts, access to second-line therapy generally required out-of-pocket payment; however, CMT remains widely accepted in Asian settings and is relatively affordable, with some regimens partially reimbursed. Therefore, in our cohort, the most common reason for not receiving second-line treatment was clinical deterioration. Despite its limitations, patients who underwent second-line CMT after first-line TKI or IO/targeted therapy had longer OS than those who received only one line of treatment. Patients who received two or more lines of systemic therapy experienced significantly improved survival, with OS extending to 40.7 months in those treated with four lines. However, no statistically significant differences in OS were observed between the different subsequent treatments, which is consistent with previous real-world reports [27,28]. Collectively, these data suggest that patient selection and access to multiple treatment lines are more critical determinants of survival than the specific agents used. Thus, CMT remains a viable option in settings where access to TKIs or IO is limited.

Several clinical factors were found to be independently associated with poor survival, including PVTT, ascites, elevated bilirubin level, high AFP level, and poor ECOG PS, which is consistent with the results of previous studies [29]. However, the extent of PVTT (main trunk vs. branch involvement) was not consistently documented in our retrospective dataset and therefore was analyzed only as a binary variable (present vs. absent), which may underestimate its prognostic heterogeneity. The number of systemic treatment lines was a strong prognostic factor for OS, emphasizing the importance of maintaining patients in a suitable condition for ongoing therapy [30]. Our findings demonstrate that the ability to receive multiple lines of systemic therapy, including CMT in later lines, was one of the strongest determinants of prolonged survival. This effect possibly reflects not only the cumulative benefit of sequential regimens, but also the preservation of patient fitness and liver function throughout the disease course. These observations underscore the importance of early supportive care, proactive toxicity management, and close multidisciplinary monitoring to maintain treatment eligibility over time. In resource-limited settings, where treatment sequencing options are constrained, preserving the opportunity for multiple lines of therapy may be as critical as the choice of the individual agents used. Previous studies have shown that survival is influenced more by liver function, PS, and sequential treatment than the initial therapy [30]. Overweight status was associated with better survival. This trend was also observed in other malignancies [31,32]. However, this association should be interpreted with caution, as it may reflect that patients with better general health and preserved nutritional status were more likely to tolerate multiple lines of therapy, rather than a direct protective effect of BMI. Residual confounding by PS, comorbidities, and liver function cannot be excluded. Collectively, these findings suggest that clinical features and liver function, rather than the specific choice of first- or second-line regimen, are the primary determinants of OS.

ORRs were generally low across all first-line regimens, with IO/targeted therapies having the highest rate (16.7%). TKIs and CMT demonstrated similar ORRs of approximately 9–10%. Consistent with earlier findings, lenvatinib had a higher ORR than sorafenib [6,7]; however, the observed ORR for IO monotherapy in this study must be interpreted cautiously, as it was based on a single patient who also underwent EBRT and TACE subsequently. These results suggest that IO/targeted therapy may be preferable in patients for whom tumor response is a priority [26,33]. Additionally, the ORR for CMT (including FOLFOX therapy) was 10%, supporting its role as an acceptable option when standard therapies are not feasible. Notably, CR was most common in patients who underwent combined-modality treatment, such as EBRT or TACE, highlighting the value of multidisciplinary management in advanced HCC [34].

The strengths of this study include the large sample size, long observation period covering evolving treatment paradigms, and detailed treatment-sequence analysis. Furthermore, it reflects real-world scenarios in resource-limited settings where treatment decisions are influenced by both clinical and socioeconomic factors. The study was not designed to compare efficacy across regimens but to describe real-world treatment patterns and their associated survival outcomes. Nonetheless, some limitations of this study must be acknowledged. The retrospective design may have introduced selection bias, and the treatment regimen heterogeneity and small subgroup sizes limit the ability to draw definitive conclusions regarding comparative efficacy. Considering the single-center retrospective design and potential selection bias, our findings may not fully represent treatment patterns and survival outcomes across all low-resource settings. Multi-center studies with larger and more diverse patient populations are warranted to confirm the generalizability of these results. Future studies should comprehensively analyze side effects, quality of life, and cost-effectiveness to better guide treatment selection beyond survival outcomes.

## 5. Conclusions

This study highlights the complexity of systemic treatment selection and sequencing in the context of advanced HCC, particularly in real-world settings where access to novel therapies is limited. Although IO-based combinations were associated with improved outcomes in clinical trials, TKIs and CMT remain essential options in clinical practice. The specific regimens used in subsequent lines may be less important than the overall ability to administer multiple lines of therapy, which was found to be strongly associated with prolonged survival. Our findings emphasize the importance of early supportive care to preserve treatment eligibility and that the involvement of a multidisciplinary team is vital for optimizing clinical status and tailoring locoregional interventions for selected patients. Thus, the real-world evidence from this study could help inform future treatment strategies and guideline development for advanced HCC in diverse healthcare contexts.

## Figures and Tables

**Table 1:** Baseline characteristics.

	TKI (*n* = 230)	CMT (*n* = 77)	IO/Targeted Therapy (*n* = 12)	Dual IO (*n* = 6)	IO-Monotherapy (*n* = 5)	Total (*n* = 330)	*p*-Value
First-line regimen, *n* (%)							<0.001
Sorafenib	162 (70.4)	-	-	-	-	162 (49.1)	
Lenvatinib	68 (29.6)	-	-	-	-	68 (20.6)	
FOLFOX	-	66 (85.7)	-	-	-	66 (20)	
Doxorubicin	-	11 (14.3)	-	-	-	11 (3.3)	
Atezolizumab/bevacizumab	-	-	12 (100)	-	-	12 (3.6)	
Durvalumab/tremelimumab	-	-	-	6 (100)	-	6 (1.8)	
Nivolumab	-	-	-	-	5 (100)	5 (1.5)	
Age, years (SD)	64.6 (10.2)	56.4 (9.8)	63.6 (11.1)	61.1 (8.1)	64.6 (13.3)	62.6 (10.7)	<0.001
<65 years, *n* (%)	124 (53.9)	64 (83.1)	6 (50.0)	4 (66.7)	2 (40.0)	200 (60.6)	
≥65 years, *n* (%)	106 (46.1)	13 (16.9)	6 (50.0)	2 (33.3)	3 (60.0)	130 (39.4)	
Sex, *n* (%)							0.6
Female	52 (22.6)	13 (16.9)	2 (16.7)	1 (16.7)	2 (40)	70 (21.2)	
Male	178 (77.4)	64 (83.1)	10 (83.3)	5 (83.3)	3 (60)	260 (78.8)	
BMI, *n* (%)							0.522
<18.5 kg/m^2^	30 (13)	11 (14.3)	0 (0)	0 (0)	0 (0)	41 (12.4)	
18.5–22.9 kg/m^2^	88 (38.3)	35 (45.5)	3 (25)	2 (33.3)	3 (60)	131 (39.7)	
≥23 kg/m^2^	112 (48.7)	31 (40.3)	9 (75)	4 (66.7)	2 (40)	158 (47.9)	
ECOG PS, *n* (%)							0.081
0–1	216 (93.9)	72 (93.5)	12 (100)	5 (83.3)	3 (60)	308 (93.3)	
2	14 (6.1)	5 (6.5)	0 (0)	1 (16.7)	2 (40)	22 (6.7)	
Cirrhosis, *n* (%)	220 (95.7)	72 (93.5)	11 (91.7)	5 (83.3)	5 (100)	313 (94.8)	0.368
CTP class, *n* (%)							0.004
A	193 (83.9)	53 (68.8)	11 (91.7)	4 (66.7)	2 (40)	263 (79.7)	
B	37 (16.1)	24 (31.2)	1 (8.3)	2 (33.3)	3 (60)	67 (20.3)	
Etiology of cirrhosis, *n* (%)							0.012
Alcoholic	25 (10.9)	6 (7.8)	1 (8.3)	1 (16.7)	1 (20)	34 (10.3)	
HBV	117 (50.9)	52 (67.5)	9 (75)	1 (16.7)	1 (20)	180 (54.5)	
HCV	42 (18.3)	11 (14.3)	0 (0)	2 (33.3)	2 (40)	57 (17.3)	
NAFLD	36 (15.7)	3 (3.9)	1 (8.3)	1 (16.7)	1 (20)	42 (12.7)	
No cirrhosis	10 (4.3)	5 (6.5)	1 (8.3)	1 (16.7)	0 (0)	17 (5.2)	
Number of liver masses, *n* (%)							0.061
≤5	100 (43.4)	38 (49.3)	3 (25.0)	4 (66.6)	3 (60.0)	148 (44.9)	
>5	94 (40.9)	32 (41.6)	8 (66.7)	1 (16.7)	0 (0)	135 (40.9)	
Infiltrative mass	36 (15.7)	7 (9.1)	1 (8.3)	1 (16.7)	2 (40)	47 (14.2)	
Largest tumor diameter, cm (IQR)	6.7 (3.7–11.9)	10 (4.4–14.2)	7.6 (3.3–14.3)	7.6 (7.1–15.1)	4.9 (3.6–11.8)	7.2 (3.8–12.5)	0.198
BCLC staging, *n* (%)							0.414
B	42 (18.3)	9 (11.7)	3 (25)	0 (0)	1 (20)	55 (16.7)	
C	188 (81.7)	68 (88.3)	9 (75)	6 (100)	4 (80)	275 (83.3)	
Portal vein involvement, *n* (%)	126 (54.8)	45 (58.4)	7 (58.3)	3 (50)	4 (80)	185 (56.1)	0.836
Ascites, *n* (%)	18 (7.8)	7 (9.1)	1 (8.3)	2 (33.3)	3 (60)	31 (9.4)	0.005
Extrahepatic metastasis, *n* (%) *	108 (47)	36 (46.8)	4 (33.3)	4 (66.7)	0 (0)	152 (46.1)	0.201
Lung metastasis	47 (20.4)	15 (19.5)	2 (16.7)	2 (33.3)	0 (0)	66 (20)	0.81
Peritoneal metastasis	13 (5.7)	11 (14.3)	1 (8.3)	1 (16.7)	0 (0)	26 (7.9)	0.092
Adrenal metastasis	14 (6.1)	2 (2.6)	0 (0)	0 (0)	0 (0)	16 (4.8)	0.729
Bone metastasis	18 (7.8)	2 (2.6)	1 (8.3)	0 (0)	0 (0)	21 (6.4)	0.436
Other metastases	3 (1.3)	1 (1.3)	0 (0)	1 (16.7)	0 (0)	5 (1.5)	-
Number of organ metastasis, *n* (%)							0.487
1	87 (37.8)	29 (37.7)	3 (25)	2 (33.3)	0 (0)	121 (36.7)	
≥2	143 (62.2)	48 (62.3)	9 (75)	4 (66.7)	5 (100)	209 (63.3)	
Laboratory test values							
TB, mg/dL (IQR)	0.9 (0.6–1.4)	1.2 (0.7–1.9)	0.9 (0.7–1.1)	1.3 (0.9–1.9)	1.3 (0.7–1.4)	1 (0.6–1.5)	0.078
Albumin, g/dL (SD)	3.6 (0.5)	3.4 (0.5)	3.5 (0.6)	3.2 (0.3)	3.4 (0.7)	3.5 (0.5)	0.07
AFP, ng/dL (IQR)	510 (24.7–12,043)	4222 (83.3–29,648)	17 (11.1–11,762)	11,085.3 (4548.1–22,298)	498.6 (13.4–34,127)	725 (27.7–1878)	0.094
Previous treatment, *n* (%) *							
Resection	28 (12.2)	8 (10.4)	1 (8.3)	0 (0)	0 (0)	37 (11.2)	0.988
Ablative treatment	41 (17.8)	7 (9.1)	2 (16.7)	2 (33.3)	1 (20)	53 (16.1)	0.188
TACE	104 (45.2)	28 (36.4)	4 (33.3)	3 (50)	3 (60)	142 (43)	0.54

* More than one answer possible. TKI, tyrosine kinase inhibitor; CMT, chemotherapy; IO, immunotherapy; FOLFOX, 5-fluorouracil, leucovorin, and oxaliplatin; SD, standard deviation; IQR, interquartile range; BMI, body mass index; ECOG, Eastern Cooperative Oncology Group; PS, performance status; CTP class, Child–Turcotte–Pugh class; HBV, hepatitis B virus; HCV, hepatitis C virus; NAFLD, nonalcoholic fatty liver disease; BCLC, Barcelona clinic liver cancer; TB, total bilirubin; AFP, alpha-fetoprotein; TACE, trans-arterial chemoembolization.

**Table 2:** Overall survival for each treatment pattern.

First-Line Treatment	Second-Line Treatment	*n*	OS	HR (95% CI)	*p* Value
First-line TKI cohort					
TKI	-	168	4.67	Ref	
TKI	TKI	13	33.07	0.32 (0.17–0.59)	<0.001
TKI	IO	30	18.53	0.41 (0.26–0.63)	<0.001
TKI	CMT	18	13.08	0.55 (0.32–0.94)	0.028
TKI	IO/targeted therapy	1	NA *	NA	NA
First-line CMT cohort					
CMT	-	63	3.48	Ref	
CMT	TKI	3	12.32	0.38 (0.09–1.57)	0.181
CMT	IO	1	5.82	1.07 (0.15–7.77)	0.950
CMT	CMT	10	15.56	0.48 (0.24–0.94)	0.034
First-line IO/targeted therapy cohort					
IO/targeted therapy	-	7	3.48	Ref	
IO/targeted therapy	TKI	2	11.63	0.59 (0.07–5.21)	0.636
IO/targeted therapy	IO	2	5.22	0.42 (0.05–3.49)	0.420
IO/targeted therapy	CMT	1	10.97	1.22 (0.13–11.20)	0.858
First-line dual IO cohort					
Dual IO	-	1	4.73	Ref	
Dual IO	TKI	2	16.64	0.25 (0.01–5.90)	0.386
Dual IO	CMT	3	10.71	0.21 (0.01–3.90)	0.298
First-line IO-monotherapy cohort					
IO	-	2	0.62	Ref	
IO	IO/targeted therapy	1	NA **	NA	NA
IO	TKI	2	22.82	3.66^−10^ (0–inf)	0.99

* Duration of follow-up was 7.72 months. ** Duration of follow-up was 47.86 months. TKI, tyrosine kinase inhibitor; CMT, chemotherapy; IO, immunotherapy; OS, overall survival; HR, hazard ratio; CI, confidence interval; NA, not available.

**Table 3:** Prognostic factors for overall survival.

Factors	Univariate Analysis Results		Multivariate Analysis Results	
	HR (95% CI)	*p*-Value	HR (95% CI)	*p*-Value
First-line treatment				
TKI	Ref		Ref	
IO/targeted therapy	0.81 (0.41–1.58)	0.534	1.08 (0.54–2.16)	0.826
Dual IO	0.86 (0.36–2.10)	0.749	0.58 (0.21–1.62)	0.298
IO-monotherapy	0.68 (0.25–1.83)	0.443	0.56 (0.19–1.61)	0.282
CMT	1.43 (1.09–1.89)	0.010	1.07 (0.80–1.42)	0.665
Male sex	1.20 (0.89–1.6)	0.232	-	-
Age ≥ 65 years	0.95 (0.75–1.21)	0.658	-	-
BMI				
<18.5 kg/m^2^	1.33 (0.92–1.92)	0.129	1.10 (0.74–1.65)	0.624
18.5–22.9 kg/m^2^	Ref		Ref	
≥23.0 kg/m^2^	0.80 (0.62–1.02)	0.077	0.73 (0.56–0.94)	0.017
ECOG PS				
0–1	Ref			
2	1.94 (1.22–3.10)	0.006	1.76 (1.06–2.91)	0.028
Cirrhosis				
HBV	2.11 (1.11–4.00)	0.022	2.08 (0.98–4.44)	0.058
HCV	1.50 (0.75–2.98)	0.248	1.70 (0.82–3.54)	0.153
NAFLD	2.09 (1.03–4.23)	0.040	1.87 (0.89–3.94)	0.101
Alcoholic	2.54 (1.24–5.17)	0.011	2.08 (0.98–4.44)	0.058
None	Ref		Ref	
Largest tumor diameter				
<5 cm	0.53 (0.40–0.68)	<0.001	0.78 (0.58–1.03)	0.078
≥5 cm	Ref		Ref	
Extrahepatic metastasis	1.02 (0.81–1.29)	0.847	-	-
PVTT	1.91 (1.5–2.43)	<0.001	1.47 (1.13–1.92)	0.005
Ascites	2.94 (1.98–4.35)	<0.001	2.20 (1.41–3.43)	<0.001
Total bilirubin ≥ 2 mg/dL	2.87 (2.05–4.02)	<0.001	2.56 (1.77–3.71)	<0.001
Albumin ≥ 3.5 g/dL	0.54 (0.42–0.69)	<0.001	0.69 (0.53–0.89)	0.005
AFP ≥ 200 ng/dL	1.90 (1.41–2.30)	<0.001	1.73 (1.32–2.27)	<0.001
Number of lines of treatment				
1	Ref			
2	0.48 (0.35–0.66)	<0.001	0.51 (0.36–0.72)	<0.001
3	0.36 (0.21–0.62)	<0.001	0.46 (0.27–0.80)	0.005
4	0.20 (0.09–0.47)	<0.001	0.27 (0.12–0.65)	0.003

TKI, tyrosine kinase inhibitor; CMT, chemotherapy; IO, immunotherapy; BMI, body mass index; ECOG, Eastern Cooperative Oncology Group; PS, performance status; HBV, hepatitis B virus; HCV, hepatitis C virus; NAFLD, nonalcoholic fatty liver disease; PVTT, portal vein tumor thrombus; AFP, alpha-fetoprotein; HR, hazard ratio; CI, confidence interval.

## Data Availability

The original contributions presented in this study are included in the article/Supplementary Material. Further inquiries can be directed to the corresponding author.

## Supplementary Materials

The following supporting information can be downloaded at: https://www.mdpi.com/article/10.3390/cancers17172729/s1, Figure S1: OS for each first-line regimen; Figure S2: OS for each treatment pattern in the first-line TKI cohort; Figure S3: OS for each treatment pattern in the first-line CMT cohort; Figure S4: OS of CTP class A patients; Figure S5: OS of CTP class B patients; Figure S6: PFS for each first-line treatment; Figure S7: PFS of CTP class A patients; Figure S8: PFS of CTP class B patients; Table S1: Treatment information in each first-line treatment; Table S2: Systemic treatment sequence and overall survival; Table S3: Details of patients survived beyond 5 years; Table S4: PFS for each first-line regimen; Table S5: Response rate for each first-line treatment; Table S6: Response rate for each first-line TKI and CMT regimen.

## Abbreviations

The following abbreviations are used in this manuscript:

HCC	hepatocellular carcinoma
OS	overall survival
PFS	progression-free survival
ORR	objective response rate
TKI	tyrosine kinase inhibitor
CMT	chemotherapy
IO	immunotherapy
PVTT	portal vein tumor thrombus
ECOG PS	Eastern Cooperative Oncology Group performance status
BMI	body mass index
BCLC	Barcelona Clinic Liver Cancer
AFP	alpha-fetoprotein
IV	intravenously
RECIST	Response Evaluation Criteria in Solid Tumors
IQR	interquartile range
SD	standard deviation
CTP	Child–Turcotte–Pugh
CR	complete response
TACE	transarterial chemoembolization
EBRT	external beam radiotherapy
CSMBS	Civil Servant Medical Benefit Scheme
