# Supplementary material for: Real-World Systemic Treatment Patterns, Survival Outcomes, and Prognostic Factors in Advanced Hepatocellular Carcinoma: A 15-Year Experience from a Low-Resource Setting
Source: Cancers (Basel). 2025 Aug 22;17(17):2729. doi: 10.3390/cancers17172729 (PMC12427224; doi:10.3390/cancers17172729)
Supplement: Supplementary file 1 [file cancers-17-02729-s001.zip › cancers-3817457-supplementary.pdf]

**Supplementary Document**

**Supplementary Table S1** Treatment information in each first-line treatment

| | TKI (n = 230) | CMT (n = 77) | IO/targeted (n = 12) | Dual IO (n = 6) | IO (n = 5) |
|---|---|---|---|---|---|
| Number of lines of treatment, n (%) | | | | | |
| 1 | 168 (73.0) | 63 (81.8) | 7 (58.4) | 1 (16.7) | 2 (40.0) |
| 2 | 42 (18.3) | 11 (14.3) | 3 (25.0) | 4 (66.6) | 3 (60.0) |
| 3 | 14 (6.1) | 3 (3.9) | 1 (8.3) | 0 (0) | 0 (0) |
| 4 | 6 (2.6) | 0 (0) | 1 (8.3) | 1 (16.7) | 0 (0) |
| First-line treatment, n | 230 | 77 | 12 | 6 | 5 |
| Number of cycles, (IQR) | 3 (1,6) | 3 (2,6) | 5 (1.8,11.8) | 9.5 (2.8,16.5) | 9 (2,10) |
| Dose reduction, n (%) | 66 (28.7) | 58 (75.3) | 0 (0) | 0 (0) | 0 (0) |
| Discontinuation, n (%) | | | | | |
| Progressive disease | 110 (48.0) | 35 (45.5) | 8 (66.7) | 5 (83.3) | 3 (60.0) |
| Liver decompensation | 48 (21.0) | 16 (20.8) | 0 (0) | 0 (0) | 0 (0) |
| Declined performance status | 7 (3.1) | 8 (10.4) | 0 (0) | 0 (0) | 0 (0) |
| Death | 28 (12.2) | 9 (11.7) | 3 (25.0) | 1 (16.7) | 1 (20.0) |
| Prefer | 1 (0.4) | 6 (7.8) | 0 (0) | 0 (0) | 0 (0) |
| Loss of follow-up | 7 (3.1) | 1 (1.3) | 0 (0) | 0 (0) | 0 (0) |
| Adverse events | 11 (4.8) | 1 (1.3) | 0 (0) | 0 (0) | 0 (0) |
| Complete response | 2 (0.9) | 0 (0) | 0 (0) | 0 (0) | 1 (20.0) |
| Transplant | 1 (0.4) | 0 (0) | 0 (0) | 0 (0) | 0 (0) |
| Ongoing treatment | 14 (6.1) | 1 (1.3) | 1 (8.3) | 0 (0) | 0 (0) |
| Second-line treatment, n (%) | 62 (26.9) | 14 (18.2) | 5 (41.7) | 5 (83.3) | 3 (60.0) |
| Regimen, n (%) | | | | | |
| TKI | 13 (21.0) | 3 (21.4) | 2 (40.0) | 2 (40.0) | 2 (66.7) |
| IO | 30 (48.4) | 1 (7.1) | 2 (40.0) | 0 (0) | 0 (0) |
| IO/targeted therapy | 1 (1.6) | 0 (0) | 0 (0) | 0 (0) | 1 (33.3) |
| CMT | 18 (29.0) | 10 (71.4) | 1 (20.0) | 3 (60.0) | 0 (0) |
| Number of cycles, (IQR) | 4 (2,8) | 5 (3,5.8) | 3.5 (2.8,9.2) | 3 (1.8,4.5) | 8 (6,8.5) |
| Dose reduction, n (%) | 13 (21.0) | 4 (28.6) | 2 (40.0) | 2 (40.0) | 1 (33.3) |
| Discontinuation, n (%) | | | | | |
| Progressive disease | 38 (61.3) | 10 (71.4) | 3 (60.0) | 2 (40.0) | 0 (0) |
| Liver decompensation | 7 (11.3) | 1 (7.1) | 1 (20.0) | 0 (0) | 0 (0) |
| Declined performance status | 2 (3.2) | 0 (0) | 0 (0) | 0 (0) | 1 (33.3) |
| Death | 3 (4.8) | 2 (14.3) | 0 (0) | 2 (40.0) | 0 (0) |
| Patient preference | 2 (3.2) | 0 (0) | 0 (0) | 0 (0) | 0 (0) |
| Loss of follow-up | 0 (0) | 1 (7.1) | 0 (0) | 0 (0) | 0 (0) |
| Adverse events | 1 (1.6) | 0 (0) | 0 (0) | 0 (0) | 1 (33.3) |
| Complete response | 1 (1.6) | 0 (0) | 0 (0) | 0 (0) | 0 (0) |
| Ongoing treatment, (%) | 7 (11.3) | 0 (0) | 1 (20.0) | 1 (20.0) | 1 (33.3) |
| Third-line treatment, n (%) | 20 (8.7) | 3 (3.9) | 2 (16.7) | 1 (16.7) | 0 (0) |
| Regimen, n (%) | | | | | - |
| TKI | 8 (40.0) | 0 (0) | 2 (100) | 1 (100) | |

| | | | | | |
|---|---|---|---|---|---|
| IO<br>IO/TKI<br>CMT | 5 (25.0)<br>1 (5.0)<br>6 (30.0) | 1 (33.3)<br>0 (0)<br>2 (66.7) | 0 (0)<br>0 (0)<br>0 (0) | 0 (0)<br>0 (0)<br>0 (0) | |
| Number of cycles, (IQR) | 6 (2,9) | 4 (4,5) | 4.5 (3.25,5.75) | 3 (1.8,4.5) | - |
| Dose reduction, n (%) | 6 (30.0) | 2 (66.7) | 1 (50.0) | 0 (0) | - |
| Discontinuation, n (%)<br>Progressive disease<br>Liver decompensation<br>Declined performance status<br>Death | <br>14 (70.0)<br>3 (15.0)<br>2 (10.0)<br>1 (5.0) | <br>2 (66.7)<br>0 (0)<br>1 (33.3)<br>0 (0) | <br>1 (50.0)<br>0 (0)<br>0 (0)<br>0 (0) | <br>1 (100)<br>0 (0)<br>0 (0)<br>0 (0) | - |
| Ongoing treatment | 0 (0) | 0 (0) | 1 (50.0) | 0 (0) | - |
| Fourth-line treatment, n (%) | 6 (2.6) | 0 (0) | 1 (8.3) | 1 (16.7) | - |
| Regimen, n (%)<br>TKI<br>IO<br>CMT | <br>1 (16.7)<br>0 (0)<br>5 (83.3) | - | <br>1 (100)<br>0 (0)<br>0 (0) | <br>0 (0)<br>1 (100)<br>0 (0) | - |
| Number of cycles, (IQR) | 13.5 (8.25,16.5) | - | 1 | 1 | - |
| Dose reduction, n (%) | 4 (66.7) | - | 0 | 0 | - |
| Discontinuation, n (%)<br>Progressive disease<br>Liver decompensation<br>Patient preference | <br>4 (66.7)<br>1 (16.6)<br>1 (16.6) | - | <br>1 (100)<br>0 (0)<br>0 (0) | <br>0 (0)<br>1 (100)<br>0 (0) | - |

TKI, tyrosine kinase inhibitor; CMT, chemotherapy; IO, immunotherapy; IQR, interquartile range.

**Supplementary Table S2** Systemic treatment sequence and overall survival

| First-line | Second-line | Third-line | Fourth-line | N | mOS (months) |
|---|---|---|---|---|---|
| TKI | TKI | IO/Targeted therapy | CMT | 1 | NA (81.34)* |
| IO | IO/Targeted therapy | | | 1 | NA (47.86)* |
| TKI | IO | TKI | CMT | 1 | NA (46.65)* |
| TKI | IO/Targeted therapy | | | 1 | NA (7.72)* |
| IO/Targeted therapy | IO | TKI | | 1 | NA (5.22)* |
| TKI | TKI | IO | TKI | 1 | 56.11 |
| TKI | TKI | IO | CMT | 1 | 47.83 |
| TKI | IO | CMT | CMT | 1 | 33.61 |
| TKI | CMT | CMT | | 1 | 32.46 |
| TKI | CMT | CMT | CMT | 1 | 32.46 |
| Dual IO | TKI | TKI | IO | 1 | 30.36 |
| TKI | TKI | IO | | 2 | 26.53 |
| TKI | IO | TKI | | 6 | 25.81 |
| IO | TKI | | | 2 | 22.82 |
| TKI | CMT | TKI | | 1 | 20.27 |
| CMT | CMT | | | 9 | 16.79 |
| TKI | TKI | | | 8 | 16.69 |
| TKI | IO | CMT | | 3 | 16.52 |
| TKI | IO | | | 19 | 14.19 |
| TKI | CMT | IO | | 1 | 13.08 |
| CMT | TKI | CMT | | 2 | 12.32 |
| IO/Targeted therapy | TKI | | | 2 | 11.63 |
| IO/Targeted therapy | CMT | TKI | TKI | 1 | 10.97 |
| Dual IO | CMT | | | 3 | 10.71 |
| TKI | CMT | | | 14 | 10.18 |
| CMT | CMT | IO | | 1 | 9.56 |
| CMT | IO | | | 1 | 5.82 |
| IO/Targeted therapy | IO | | | 1 | 5.22 |
| Dual IO | | | | 1 | 4.73 |
| TKI | | | | 168 | 4.67 |
| CMT | TKI | | | 1 | 4.17 |
| CMT | | | | 63 | 3.48 |
| IO/Targeted therapy | | | | 7 | 3.48 |
| Dual IO | TKI | | | 1 | 2.92 |
| IO | | | | 2 | 0.62 |

* Duration of the follow-up period
OS, overall survival; TKI, tyrosine kinase inhibitor; CMT, chemotherapy; IO, immunotherapy.

**Supplementary Table S3** Details of patients survived beyond 5 years.

| Case | Age/Sex | ECOG PS | Etiology | CTP | BCLC | Disease status at systemic treatment start | First-line regimen (duration, best response) | Subsequent lines of therapy (duration, best response) | Outcome at data cut-off |
|---|---|---|---|---|---|---|---|---|---|
| 1 | 49/M | 1 | HBV cirrhosis | A(5) | B | TACE-refractory, 2 new liver lesions (largest diameter 2.9 cm) | Sorafenib (77.7 mo, PR) | None (still ongoing sorafenib) | Alive, no progression |
| 2 | 56/F | 1 | HCV cirrhosis | A(5) | C | Single lesion 3 cm, PVTT, no metastases | Sorafenib (22.1 mo, PR) | Nivolumab (32.0 mo, CR), then surveillance | Alive, no recurrence |
| 3 | 55/M | 0 | HCV cirrhosis | A(5) | C | Lung & bone mets after prior liver resection | Sorafenib (19.6 mo, SD) | Regorafenib (7.6 mo, SD) → Atezolizumab/Bevacizumab (2 mo, PD) → FOLFOX (6 mo, PR), then observation | Alive |

ECOG, Eastern Cooperative Oncology Group; PS, performance status; CTP, Child–Turcotte–Pugh; BCLC, Barcelona clinic liver cancer; HBV, hepatitis B virus ;HCV, hepatitis C virus ; TACE, trans-arterial chemoembolization; PVTT, portal vein tumor thrombus; PR, partial response; SD, stable disease; CR, complete response; PD, progressive disease; FOLFOX, 5-fluorouracil, leucovorin, and oxaliplatin.

**Supplementary Table S4** PFS for each first-line regimen

| First-line regimen | mPFS (months) | HR (95% CI) | P value |
|---|---|---|---|
| Sorafenib | 3.52 | Ref | |
| Lenvatinib | 4.86 | 0.90 (0.66,1.22) | 0.494 |
| Atezolizumab/bevacizumab | 3.48 | 0.93 (0.51,1.72) | 0.828 |
| Durvalumab/tremelimumab | 6.19 | 0.85 (0.37,1.92) | 0.689 |
| Nivolumab | 2.69 | 1.05 (0.43,2.56) | 0.922 |
| FOLFOX | 3.52 | 1.22 (0.91,1.63) | 0.193 |
| Doxorubicin | 2.53 | 1.39 (0.71,2.73) | 0.342 |

PFS, progression-free survival; FOLFOX, 5-fluorouracil, leucovorin, and oxaliplatin; HR, hazard ratio; CI, confidence interval.

**Supplementary Table S5** Response rate for each first-line treatment

| | TKI (n = 230) | CMT (n = 77) | IO/targeted (n = 12) | Dual IO (n = 6) | IO (n = 5) |
|---|---|---|---|---|---|
| ORR, n (%) | 22 (9.6) | 8 (10.4) | 2 (16.7) | 0 (0) | 1 (20.0) |
| CR, n (%) | 1 (0.4) | 0 (0) | 0 (0) | 0 (0) | 1 (20.0) |
| PR, n (%) | 21 (9.1) | 8 (10.4) | 2 (16.7) | 0 (0) | 0 (0) |
| SD, n (%) | 82 (35.7) | 14 (18.2) | 4 (33.3) | 2 (33.3) | 1 (20.0) |
| PD, n (%) | 60 (26.1) | 18 (23.4) | 3 (25.0) | 3 (50.0) | 2 (40.0) |
| Not evaluated, n (%) | 66 (28.7) | 37 (48.1) | 3 (25.0) | 1 (16.7) | 1 (20.0) |

TKI, tyrosine kinase inhibitor; CMT, chemotherapy; IO, immunotherapy; ORR, objective response rate; CR, complete response; PR, partial response; SD, stable disease; PD, progressive disease.

**Supplementary Table S6** Response rate for each first-line TKI and CMT regimen

| | Sorafenib (n = 162) | Lenvatinib (n = 68) | FOLFOX (n = 66) | Doxorubicin (n = 11) |
|---|---|---|---|---|
| ORR, n (%) | 11 (6.8) | 11 (16.2) | 7 (10.6) | 1 (9.1) |
| CR, n (%) | 1 (0.6) | 0 (10) | 0 (0) | 0 (0) |
| PR, n (%) | 10 (6.2) | 11 (16.2) | 7 (10.6) | 1 (9.1) |
| SD, n (%) | 58 (35.8) | 24 (35.3) | 13 (19.7) | 1 (9.1) |
| PD, n (%) | 46 (28.4) | 14 (20.6) | 14 (21.2) | 4 (36.4) |
| Not evaluated, n (%) | 47 (29.0) | 19 (27.9) | 32 (48.5) | 5 (45.5) |

TKI, tyrosine kinase inhibitor; CMT, chemotherapy; FOLFOX, 5-fluorouracil, leucovorin, and oxaliplatin; ORR, objective response rate; CR, complete response; PR, partial response; SD, stable

**Supplementary Figure S1** OS for each first-line regimen

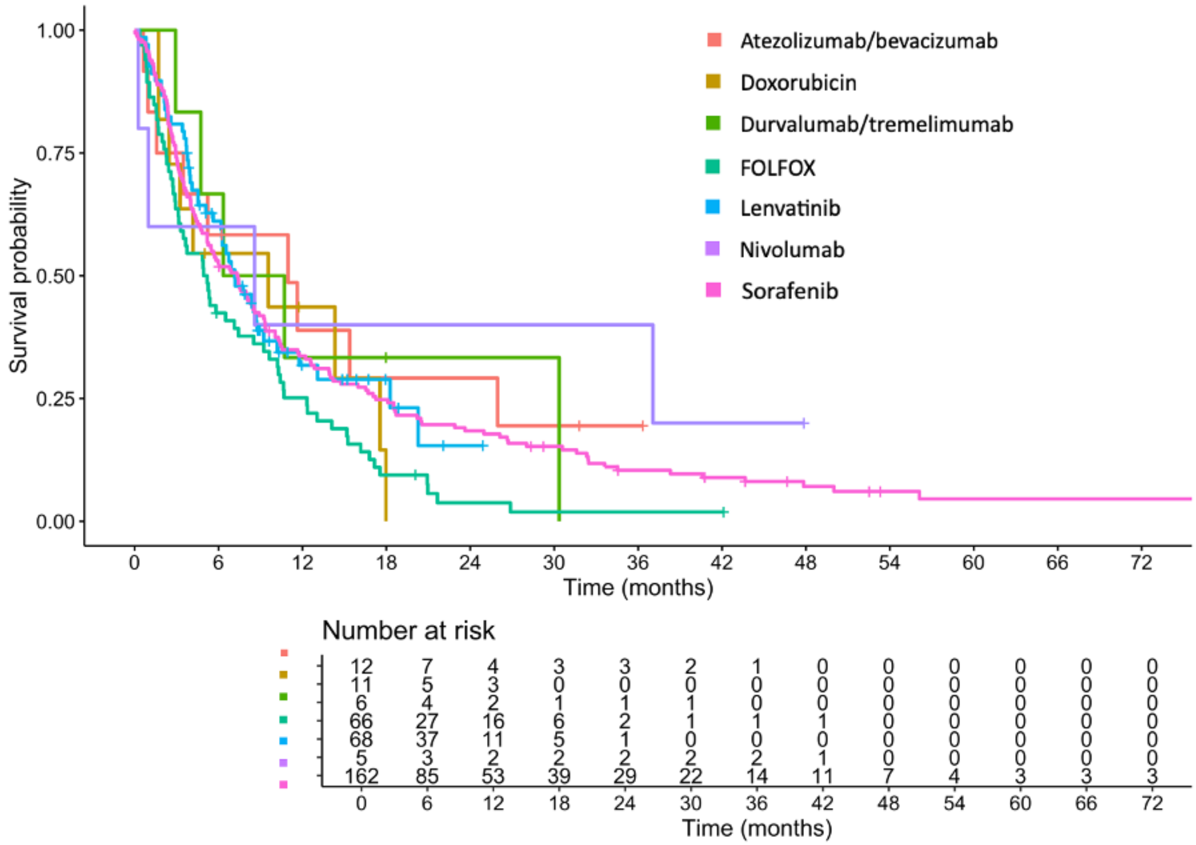


| First-line regimen | mOS (months) | HR (95% CI) | P value |
|---|---|---|---|
| Sorafenib | 7.3 | Ref | |
| Lenvatinib | 7.2 | 0.99 (0.71,1.40) | 0.980 |
| Atezolizumab/bevacizumab | 10.9 | 0.81 (0.41,1.59) | 0.535 |
| Durvalumab/tremelimumab | 8.5 | 0.86 (0.35,2.11) | 0.748 |
| Nivolumab | 8.6 | 0.68 (0.25,1.83) | 0.444 |
| FOLFOX | 5.1 | 1.48 (1.10,2.00) | 0.010 |
| Doxorubicin | 9.6 | 1.17 (0.59,2.29) | 0.657 |

OS, overall survival; FOLFOX, 5-fluorouracil, leucovorin, and oxaliplatin; HR, hazard ratio; CI, confidence interval.

**Supplementary Figure S2** OS for each treatment pattern in the first-line TKI cohort

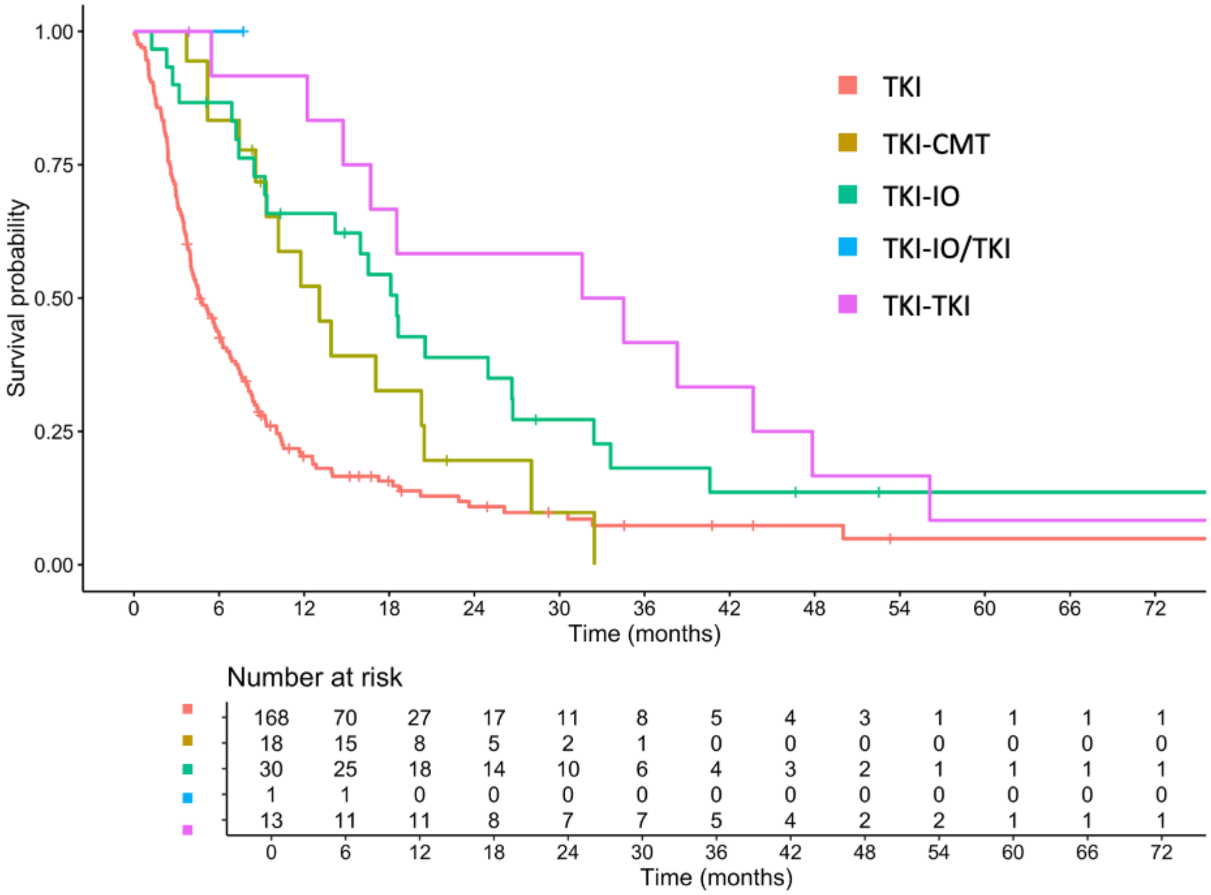

**Supplementary Figure S3** OS for each treatment pattern in the first-line CMT cohort

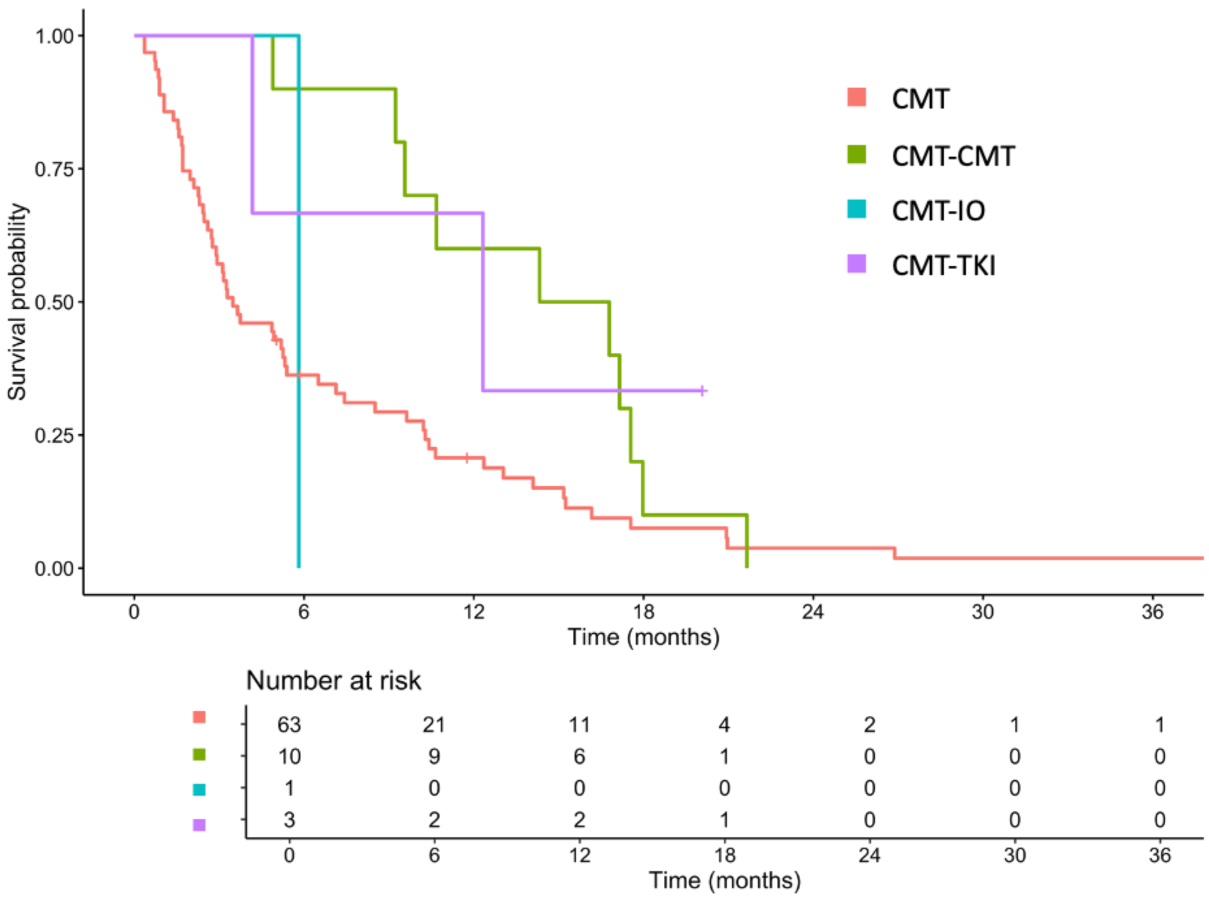

**Supplementary Figure S4** OS of CTP class A patients

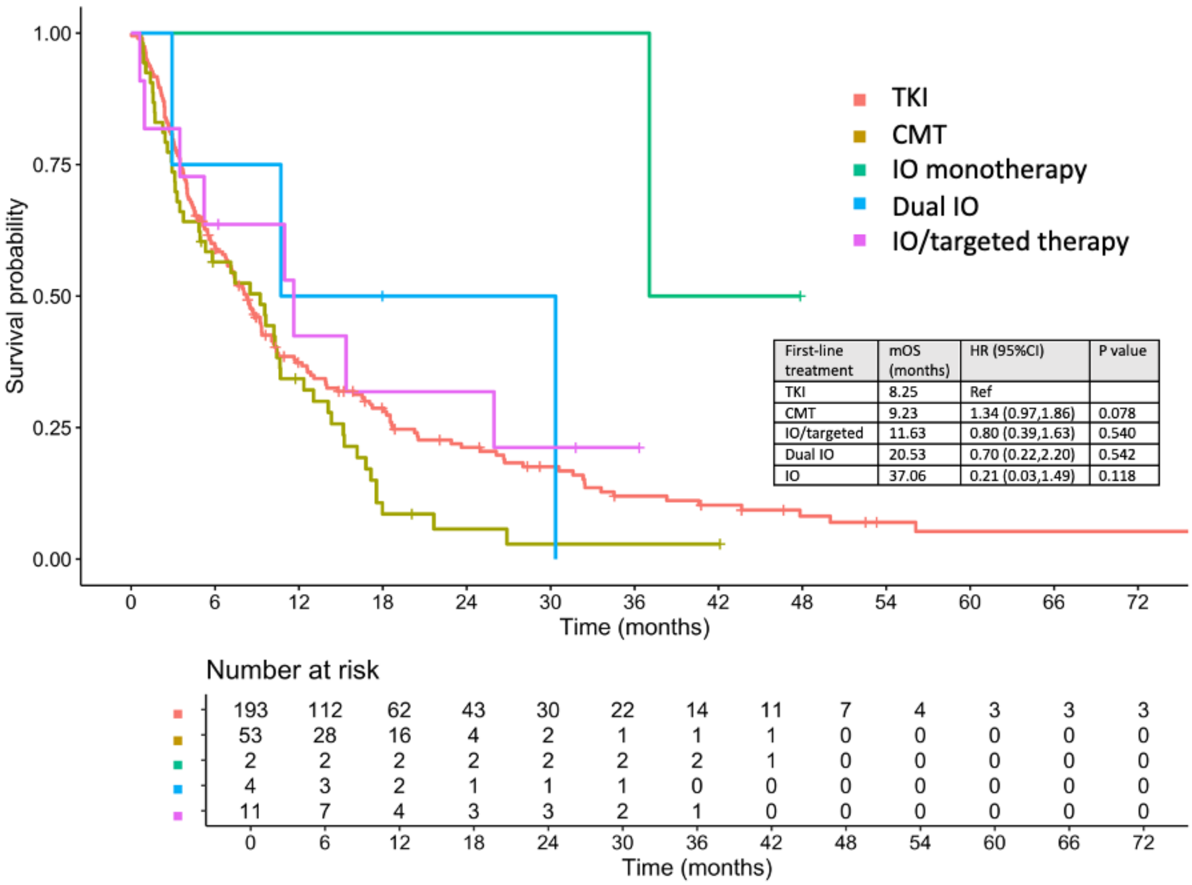


| First-line treatment | mOS (months) | HR (95%CI) | P value |
|---|---|---|---|
| TKI | 8.25 | Ref | |
| CMT | 9.23 | 1.34 (0.97,1.86) | 0.078 |
| IO/targeted | 11.63 | 0.80 (0.39,1.63) | 0.540 |
| Dual IO | 20.53 | 0.70 (0.22,2.20) | 0.542 |
| IO | 37.06 | 0.21 (0.03,1.49) | 0.118 |

**Supplementary Figure S5** OS of CTP class B patients

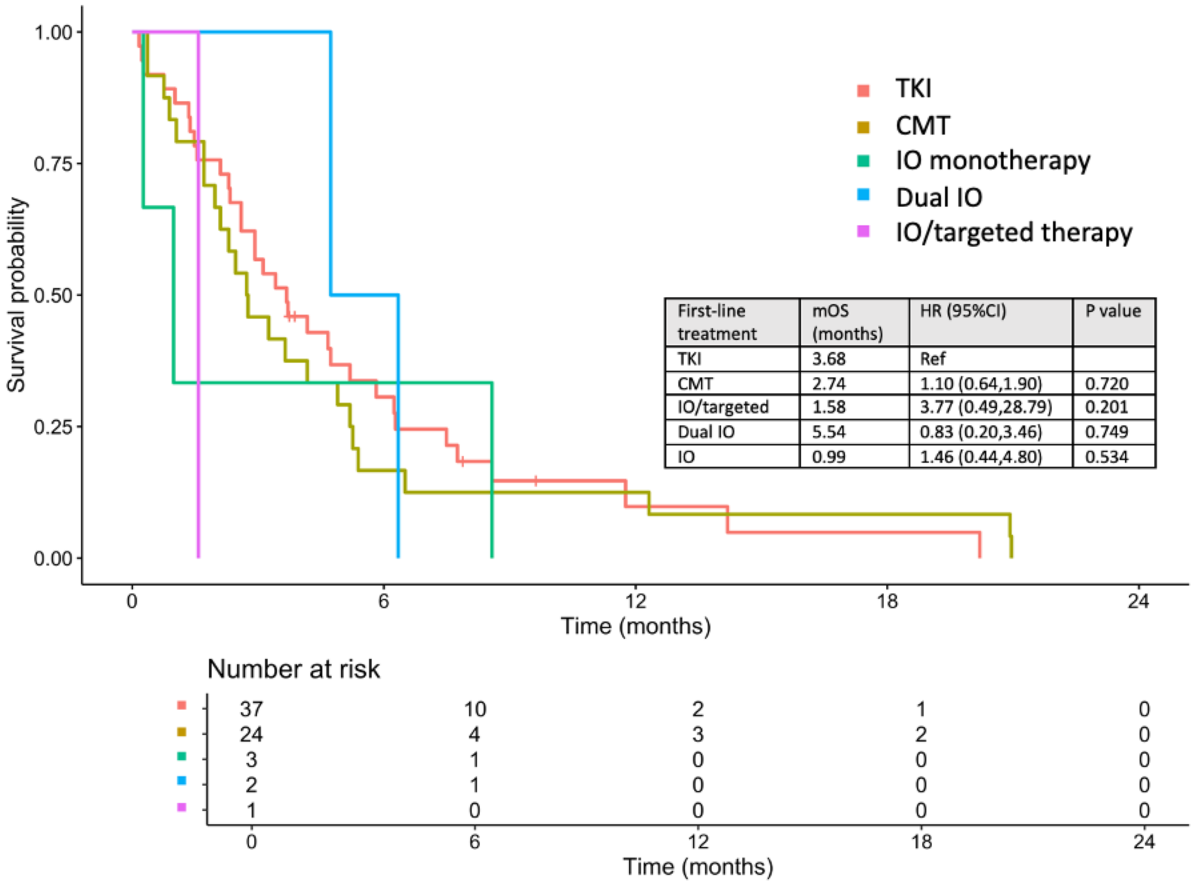

**Supplementary Figure S6** PFS for each first-line treatment

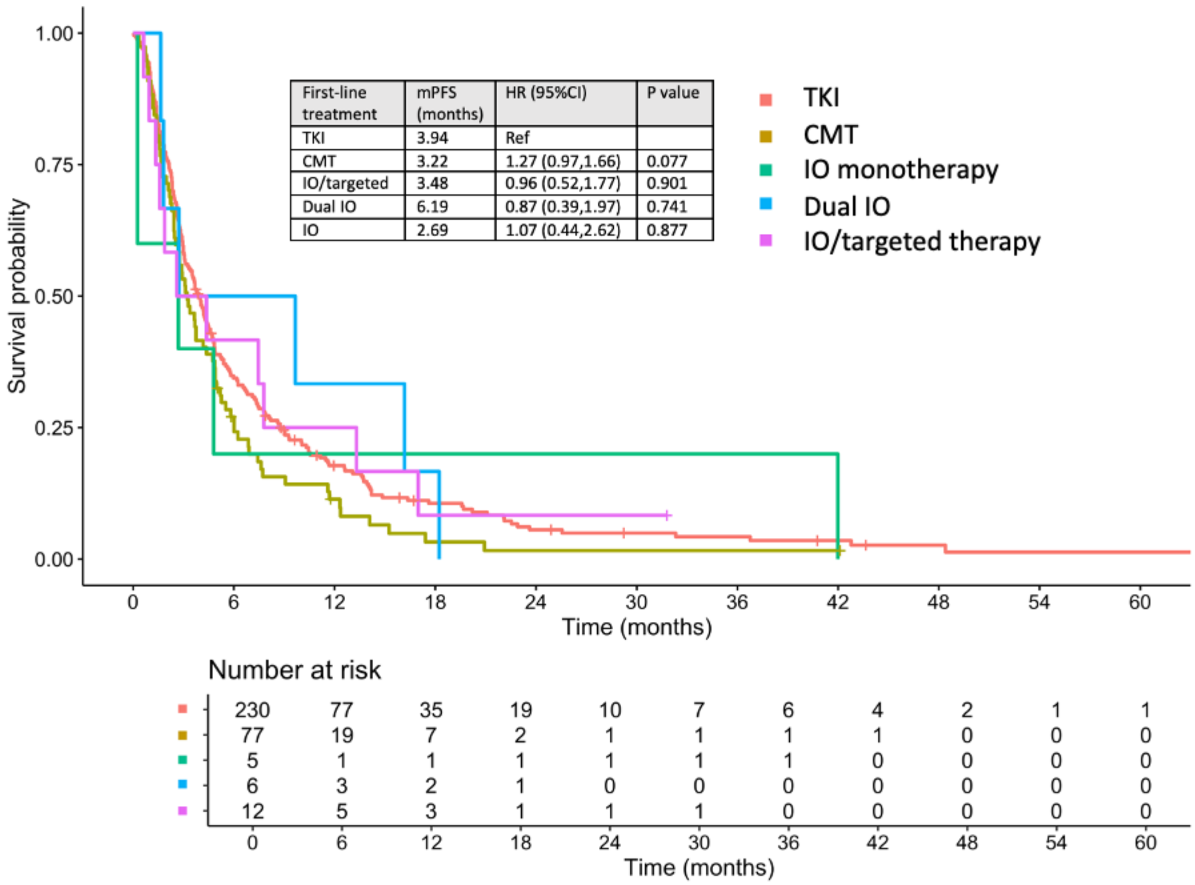

**Supplementary Figure S7** PFS of CTP class A patients

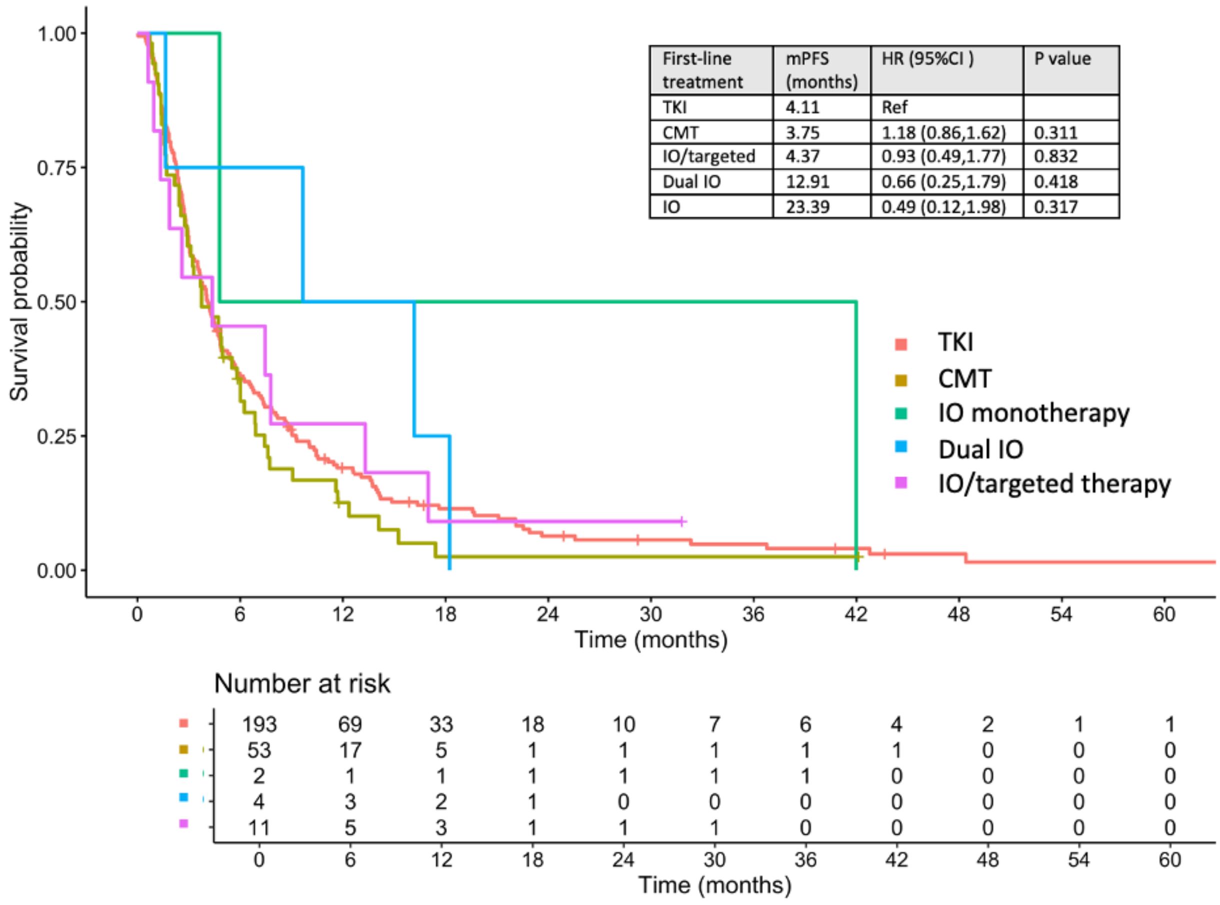

**Supplementary Figure S8** PFS of CTP class B patients

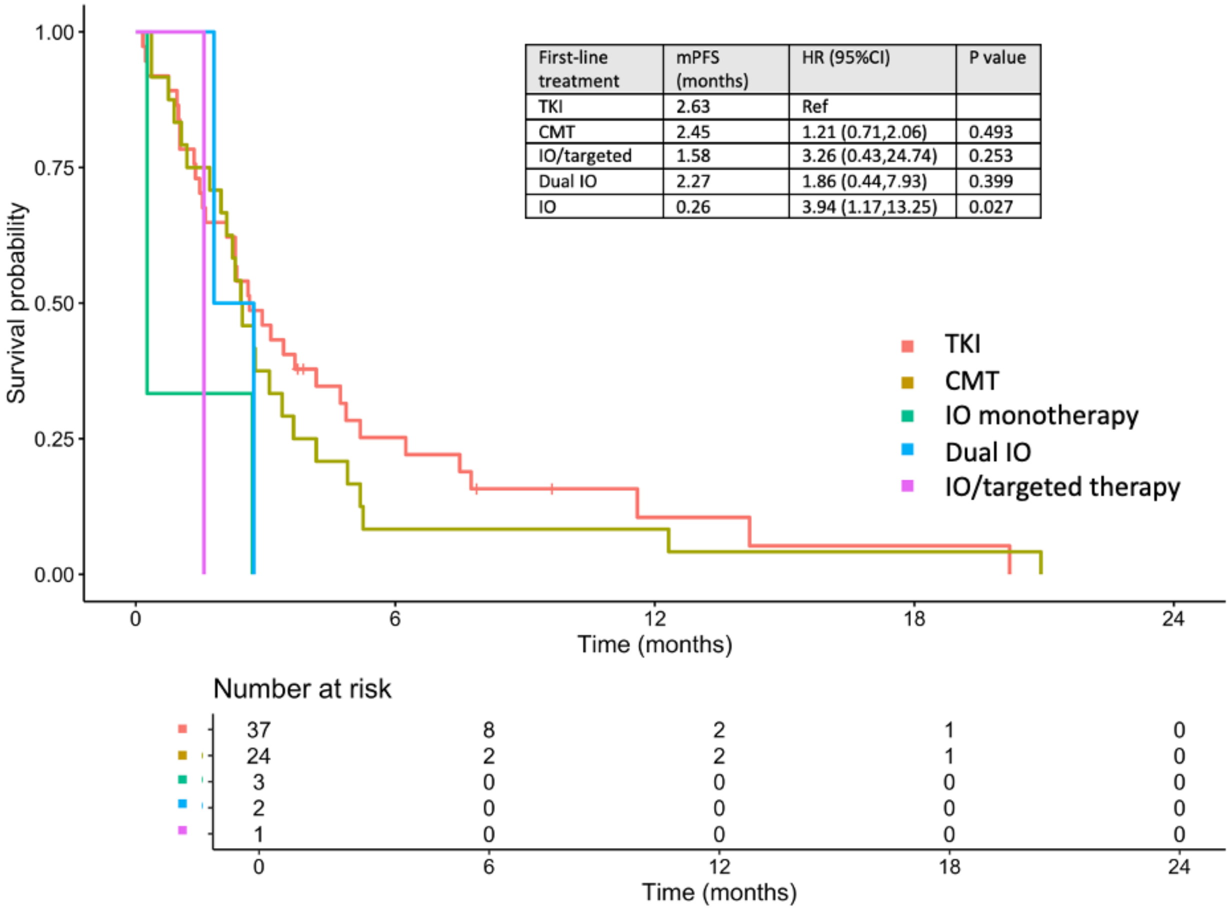


| First-line treatment | mPFS (months) | HR (95%CI) | P value |
|---|---|---|---|
| TKI | 2.63 | Ref | |
| CMT | 2.45 | 1.21 (0.71,2.06) | 0.493 |
| IO/targeted | 1.58 | 3.26 (0.43,24.74) | 0.253 |
| Dual IO | 2.27 | 1.86 (0.44,7.93) | 0.399 |
| IO | 0.26 | 3.94 (1.17,13.25) | 0.027 |
